# Is there room for allergen immunotherapy for the treatment of atopic dermatitis in the precision medicine era?

**DOI:** 10.3389/fped.2022.1050560

**Published:** 2022-11-02

**Authors:** Benedetta Pessina, Mattia Giovannini, Francesca Mori, Giuseppe Di Cara, Elio Novembre, Susan Chan, Carsten Flohr, George du Toit

**Affiliations:** ^1^ Department of Health Sciences, University of Florence, Florence, Italy; ^2^ Allergy Unit, Department of Pediatrics, Meyer Children's Hospital, Florence, Italy; ^3^ Pediatric Allergy Group, Department of Women and Children's Health, School of Life Course Sciences, King's College London, London, United Kingdom; ^4^ Section of Pediatrics, Department of Medicine and Surgery, University of Perugia, Perugia, Italy; ^5^ Pediatric Allergy Group, Peter Gorer Department of Immunobiology, School of Immunology & Microbial Sciences, King's College London, London, United Kingdom; ^6^ Children's Allergy Service, Evelina London Children's Hospital, Guy's and St Thomas’ NHS Foundation Trust, London, United Kingdom; ^7^ Unit for Pediatric & Population-Based Dermatology Research, St John's Institute of Dermatology, Guy's and St Thomas’ NHS Foundation Trust and King's College London, London, United Kingdom

**Keywords:** atopic dermatitis, allergen immunotherapy, house dust mites, precision medicine, pediatrics

## Introduction

Atopic dermatitis (AD), or atopic eczema, is an inflammatory, pruritic, chronic or chronically relapsing skin disease, often occurring in families with atopic diseases.

AD is a common non-communicable skin disease, affecting up to 20% of children (1) and 8% of adults (2). It often begins in early childhood, but about one-third of cases may develop in adulthood. The disease is mild in most affected children and may resolve before the age of 2 years old in about 40% of the cases (3). Other cases may persist into adulthood (4), e.g., more severe cases, which account for about 7% of pediatric cases (5), those with early onset or concomitant allergic sensitization (6). AD may be the first step which leads to the development of other atopic diseases, such as food allergies, allergic rhinoconjunctivitis and asthma (7). Some authors also consider eosinophilic esophagitis as a possible late-onset manifestation of this “allergic march” (8).

The core clinical manifestations of AD are skin dryness and pruritic, poorly defined eczematous lesions, which vary from acute, red and exudative papules and vesicles to subacute-chronic and often lichenified erythematous lesions. The distribution and morphology of the lesions is highly variable across patients and across different age groups (9).

Quality of life of the patients is influenced by the psychological and physical burden intrinsic to AD lesions, which can lead to sleep deprivation due to the itch and social embarrassment due to lesion visibility. These factors further promote a higher prevalence of depression and anxiety in AD patients (10). There is also a potential association with mental disorders, which is not fully understood (10–12).

Diagnosis of AD is primarily clinical (13–16). There are a number of physician-assessed severity scales, commonly used both during a first evaluation and in the follow-up of AD patients, such as the Eczema Area Severity Index (EASI) and the SCORing Atopic Dermatitis (SCORAD) Index, which are the recommended systems to measure clinical manifestations (17–19). In clinical practice, patient-assessed scores are more commonly used (e.g., the Patient-Orientated Eczema Measure – POEM) (17).

As regards pathophysiology, AD is a multifactorial disorder characterized by an impaired skin barrier and immune dysregulation, both contributing to skin inflammation (20, 21), which can be further perpetuated by superinfection with bacteria such as *Staphylococcus aureus* (22). Like many other immune-mediated disorders, AD is not a single disease entity but has several endotypes characterized by discrete immunological and molecular mechanisms (23–25). This concept is becoming relevant for disease treatment, potentially leading in the future to a more personalized approach in AD management, also regarding novel therapies.

Standard of treatment for AD consists of the regular use of emollients to preserve skin barrier function, and topical corticosteroids or calcineurin inhibitors to treat acute flares, but often also as maintenance (e.g., 2–3 times weekly) therapy. Severe or refractory cases can benefit from systemic immunomodulatory therapy, including immuno-suppressive drugs, biologics and small molecules, such as Janus kinase (JAK) inhibitors (26). A role for allergen immunotherapy (AIT) in AD management has been suggested, but clear indications for its application are still lacking (27, 28).

AIT is an allergen-based, tolerance-inducing treatment for allergic diseases, nowadays used in the management of allergic rhinitis, as well as, e.g., food and venom allergies. It consists in the repeated administration of definite quantities of allergen extracts, mainly *via* the subcutaneous (SCIT), sublingual (SLIT) (29) or oral route (OIT). AIT modifies the immune regulation of allergic responses (30), by inducing regulatory T (Treg) cells to produce, e.g., interleukin (IL)-10, transforming growth factor (TGF)-beta and IL-35 and to express surface molecules as cytotoxic T-lymphocyte-associated protein-4 (CTLA4) and programmed cell death protein-1 (PD1), all of which contribute to suppression of Th2 cells and cytokines, basophils, and eosinophils. Treg cells also induce allergen-specific regulatory B (Breg) cells (31). The suppressive milieu limits the production of IgE and induces production of IgG4 from B cells, which can act as a decoy for allergen binding (32). Breg cells, regulatory natural killer (NKreg) cells, and regulatory innate lymphoid cells (ILCregs) all contribute to the induction and maintenance of allergen-specific tolerance (33). Disease-modifying interventions which act on the pathophysiology of AD may be used as tools to prevent exacerbation of disease or progression of the atopic march, when used adequately and in the right patients.

This paper aims to provide a review of the current role of AIT in the management of AD, discussing the most recent evidence on the safety and efficacy of AIT in AD treatment and summarizing the latest international recommendations on this topic, with a focus on pediatric AD.

## Materials and methods

We performed a literature search in Medline through PubMed using default keywords related to pediatric Atopic Dermatitis and Allergen Immunotherapy. Original studies and review articles, with a focus on meta-analyses and randomized controlled trials (RCTs) in English, were identified up to May 1st, 2022.

## Results

Among the most recent meta-analyses, Bae et al. performed a systematic review and meta-analysis in 2013 to assess the efficacy of AIT for AD patients (34). The analysis included 8 studies, 7 placebo-controlled RCTs and one quasi-RCT, including a total of 385 subjects. The characteristics of the studies are summarized in Table 1A. One of the studies included a population of adults only.

**Table 1:** Summary of the studies included in systematic review and meta-analysis, (**A**) adapted from Bae et al. (34) and (**B**) adapted from Tam et al. (35), and (**C**) of the two latest RCTs, including a pediatric population. DB, double-blind; HDM, house dust mites; OL, open label; PC, placebo-controlled; SCIT, subcutaneous immunotherapy; RCT, randomized controlled trial; SLIT, sublingual immunotherapy; y.o, years old.

(+) Study, year	Study design	Participants (number, age range)	Intervention (type - allergen)	Duration
(**A**)				
Kaufman and Roth, 1974 (36)	qRCT DB PC	52, children and adults: 2–47 year old.	SCIT – HDM, animal dander, pollen, molds	24 months
Warner et al., 1978 (37)	RCT DB PC	20, children: 5–14 year old.	SCIT – HDM	12 months
Glover and Atherton, 1992 (38)	RCT DB PC	24, children: 5–16 year old.	SCIT – HDM	8 months
Leroy et al., 1993 (39)	RCT DB PC	23, children and adults: 15–64 year old.	SCIT – HDM	4 months
Galli et al., 1994 (40)	RCT PC	34, children: 0.5–12 year old.	SLIT – HDM	36 months
Silny and Czarnecka-Operacz, 2006 (41)	RCT DB PC	20, children and adults: 5–40 year old.	SCIT – HDM, animal dander, pollen	12 months
Pajno et al., 2007 (42)	RCT DB PC	56, children: 5–16 year old.	SLIT – HDM	18 months
Novak et al., 2012 (43)	RCT DB PC	168, adults: 18–66 year old.	SCIT – HDM	18 months
(**B**)				
Sanchez et al., 2012 (44)	RCT OL PC	65, children and adults: 3–25 year old.	SCIT – HDM	12 months
Luna-Pech et al., 2013 (45)	RCT DB PC	68, children: 4–10 year old.	SLIT – HDM	12 months
Qin et al., 2014 (46)	RCT PC	107, adults: 18–46 year old.	SLIT – HDM	12 months
Di Rienzo et al., 2014 (47)	RCT OL PC	57, children: 5–18 year old	SLIT – HDM	12 months
(**C**)				
Yu et al., 2021 (48)	RCT OL PC	96, children and adults: 4–60 year old.	SLIT – HDM	24 months
Langer et al., 2022 (49)	RCT DB PC	91, children and adults: 3–62 year old.	SLIT – HDM	18 months

Overall, the work demonstrated moderate-level evidence for the efficacy of AIT for AD patients, compared with placebo. Stratifying by type of intervention (SCIT vs. SLIT), a subgroup analysis study showed a conserved significant positive effect of SCIT on AD, while that of SLIT studies did not. In the subgroup analysis by age (adults vs. children), AIT did not prove significant efficacy in children. The other subgroup analyses (short-term vs. long-term treatment, mild vs. severe AD) showed significant efficacy of AIT in the six long-term treatment studies (more than one year) and in the five severe AD studies.

Comparable proportions of systemic (from 0.0% to 8.0% vs. from 0.0% to 10.7%, respectively) and local adverse reactions (from 6.3% to 80.0% vs. from 0.0% to 60.0%, respectively) were reported in the AIT and placebo groups, without fatal or near-fatal adverse events.

In conclusion, this meta-analysis provided moderate-level evidence for AIT efficacy in AD, in the general population but not in the children subgroup. However, these findings are based on a small number of RCTs, with considerable heterogeneity in study design, different allergens type and dose, age groups, administration schedules, duration of treatment and outcomes studied. Moreover, the authors do not further specify patient characteristics, and some of the trials also include patients who received systemic corticosteroids to control disease.

A second work was carried out by Tam et al. (35), who performed a Cochrane systematic review and meta-analysis in 2016 to assess the effect of AIT compared to placebo or standard treatment in AD. In this analysis, 12 RCTs were included with a total number of 733 participants. Eight of the included studies were in common with the meta-analysis performed by Bae et al. (34) and four were newly published studies, whose characteristics are summarized in Table 1B. One of the new studies was on an entirely adult population.

In this Cochrane, many different physician-assessed and patient-reported outcomes were analyzed. A significant improvement in disease severity based upon investigator- or physician-rated global assessment of SCORAD was shown. However, no significant difference in patient or parent-reported clinical manifestations of itching or sleep disturbance as assessed by SCORAD part C was observed. The overall quality of the evidence was low, mainly due to the differing results between studies and the lack of blinding in some studies. Moreover, subgroup analyses for allergen-type, patient age and disease severity could not be performed, because of the small number of trials that contributed with data to the analyses.

No statistically significant increase in the risk of local reactions between AIT and control groups was found in 484 participants. In a total of 492 participants, no statistically significant increase in the risk of systemic reactions was observed, with 18 events in the AIT group and 15 in the control group.

After the two above-mentioned meta-analyses, three new RCTs have been published, one of them on an entirely adult population (50) and two including children (48, 49), both investigating the efficacy of SLIT in subjects with AD and sensitized to house dust mites (HDM). The characteristics of the two latest RCTs, including a pediatric population, are summarized in Table 1C.

Among the two studies including children, in 2021 a RCT was published by Yu et al., on 96 HDM sensitized subjects (age 4–60 years, mean age 27 years). Seventy-seven subjects completed a full study period of 24 months, 38 receiving only standard treatment (oral antihistamines and/or topical steroid) and 39 receiving HDM SLIT (48). The patients in the treatment group showed a significant decrease from baseline SCORAD, Visual Analogue Scale (VAS) and rescue medication score from 12 months of treatment on, compared with the control group, without severe adverse events during the therapy. However, this was an unblinded study on a relatively small cohort of patients.

The latest RCT was published in February 2022 by Langer et al. and was a double-blind, placebo-controlled study on 91 patients (3–62 years, 40% < 12 years, distributed in the study arms), with SCORAD score greater than or equal to 15 and positive skin test result and/or IgE to *Dermatophagoides pteronyssinus* (49). Sixty-six patients (31 in the placebo group) completed the study and received placebo or HDM drops for 18 months. The work demonstrated a statistically significant difference in the decrease in mean SCORAD score from baseline to 18 months. There were similar reductions in objective SCORAD and an higher proportion of patients with Investigator Global Assessment 1/0, with the absence of severe adverse events. On the other hand, no difference in EASI and DLQI scores, VAS for symptoms, and pruritus scores was observed. Whilst this study demonstrates the safety and the potential efficacy of AIT in AD patients, there was a high drop-out rate (27%), according to other previous studies (20% in the above-mentioned Yu et al. paper) (48). Moreover, the results were not consistent across the different outcome measures, lending uncertainty to their interpretation.

## Discussion

The American Academy of Dermatology published its guidelines for the management of AD in 2014 (51). In their opinion, AIT could be considered as a possible adjunct to conventional therapy but, due to the small number of published studies and the conflicting evidence, AIT was not recommended for the management of disease in the general AD population.

In 2017, the Italian Society of Pediatric Allergy and Immunology summarized the evidence in their clinical practice recommendations for AIT in children, stating that existing studies were often uncontrolled and overall results were controversial (52). They acknowledged the clinical efficacy of AIT in extrinsic AD, but they were unable to state clear recommendations. They concluded that the use of AIT in AD was still largely experimental, and the indications were still limited to those AD patients with co-existing allergic rhinitis or asthma.

In 2019, the European Academy of Allergy and Clinical Immunology (EAACI) was involved in the consensus-based European guidelines for the treatment of AD, where it was stated that the evidence regarding the use of AIT in AD treatment was conflicting, with more recent literature being more favorable towards therapy, which may have positive effects in chosen, highly sensitized patients (53). In these guidelines, AIT was not recommended as a general treatment option for AD, but its use was potentially suggested in selected cases, such as in patients with house dust mite, birch or grass pollen sensitization, with severe AD, and with a history of clinical exacerbation after exposure to the causative allergen or a positive corresponding atopy patch test. Moreover, it was specified that AIT was not contraindicated in patients with concomitant respiratory allergic diseases (mild allergic bronchial asthma or allergic rhinoconjunctivitis) and AD. The European Task Force on Atopic Dermatitis (ETFAD) of the European Academy of Dermatology and Venerology (EADV) 2020 position paper reinforced the same concepts of patient selection and underlined the fact that AIT may be used in AD when approved indications for this treatment exist in the same patient, as in the case of concomitant respiratory allergic diseases (26). Finally, the most recent European Dermatology Forum in the European guidelines (EuroGuiDerm) 2022 on atopic eczema treatment recommended once more against the use of AIT as routine treatment, but suggested to consider it for selected patients with house dust mite, birch or grass pollen sensitization, and a history of clinical exacerbation after exposure to the causative allergen or a positive corresponding atopy patch test, as mentioned above (54).

In conclusion, AIT is still not usually recommended as a treatment option for AD, and its employment remains empirical in clinical practice. Recent data suggest that AIT prescription may be considered but should be individualized for each patient, evaluating the risk-benefit ratio and after discussion with the patients/parents. A more precise selection of clinical phenotypes may help identify those patients with AD who could benefit from a tailored approach to AIT, such as patients with a proven sensitization to aeroallergens, particularly house dust mites, patients who present flare-ups induced by exposure to aeroallergens, and concomitant allergic rhinitis or asthma. In the pediatric subgroup, it may be relevant to consider which patients have a lower chance of spontaneous resolution before adulthood. There is a need for more well designed and adequately powered RCTs, with standardized outcomes, to better inform recommendations for AIT use in clinical practice for patients with AD. This is particularly true in the pediatric subgroup, for which an even greater lack of studies and recommendations is observed.

## Author contributions

MG and EN conceptualized the work. BP and MG collected the data and drafted the manuscript. BP, MG, FM, GDC, EN, SC, CF and GDT analyzed the data. BP, MG, FM, GDC, EN, SC, CF and GDT critically revised the manuscript. All authors contributed to the article and approved the submitted version.
